# Prevalence of Over‐the‐Counter Drug Abuse and Associated Psychosocial Factors Among High School Students: A Nationwide Cross‐Sectional Survey in Japan

**DOI:** 10.1002/npr2.70030

**Published:** 2025-06-14

**Authors:** Takuya Shimane, Satoshi Inoura, Maki Kitamura, Kunihiko Kitagaki, Koji Tominaga, Toshihiko Matsumoto

**Affiliations:** ^1^ Department of Drug Dependence Research National Institute of Mental Health, National Center of Neurology and Psychiatry Kodaira Japan; ^2^ Department of Nursing, Faculty of Nursing Niigata Seiryo University Niigata Japan; ^3^ Faculty of Pharmaceutical Sciences Tokyo University of Pharmacy and Life Sciences Hachioji Japan; ^4^ Japan Pharmaceutical Association Shinjuku Japan

**Keywords:** adolescent, epidemiologic research design, nonprescription drugs, social isolation, substance‐related disorders

## Abstract

**Aim:**

This study estimated the prevalence of over‐the‐counter (OTC) drug abuse among high school students in Japan and clarified the predictors related to their school and home life.

**Methods:**

A nationwide cross‐sectional survey was conducted between September 2021 and March 2022. The survey included 41 357 valid responses from 202 randomly selected regular high schools in Japan. Respondents were asked about their history of OTC drug abuse within the past year, which was the primary outcome of this study. Multivariate logistic regression analyses were performed to identify the predictors of OTC drug abuse.

**Results:**

The estimated prevalence of OTC drug abuse over the past year was 1.5% (95% CI: 1.4–1.6). Dissatisfaction with school life (AOR = 2.57, 95% CI = 1.80–3.66), hours spent in a day without parents (AOR = 1.59, 95% CI = 1.27–2.00), and COVID‐19‐related stress (AOR = 1.53, 95% CI = 1.14–2.05) significantly increased the risk of OTC drug abuse. Conversely, positive extracurricular activities (AOR = 0.80, 95% CI = 0.63–1.00), close communication with the mother (AOR = 0.66, 95% CI = 0.51–0.87), and high drug‐refusal skills (AOR = 0.57, 95% CI = 0.41–0.79) significantly reduced the risk.

**Conclusions:**

OTC drug abuse is widespread among high school students in Japan, and attention should be paid to students who are isolated at school and home. Therefore, it is important to develop effective prevention, education, and treatment programs for adolescents that consider the risks and protective factors associated with OTC drug abuse.

## Abstract 2

A nationwide survey of Japanese high school students revealed the current state of over‐the‐counter drug abuse. The prevalence of over‐the‐counter drug abuse within the past year was estimated to be 1.5%. Risk and protective factors in school and home life were identified.

## Graphical abstract

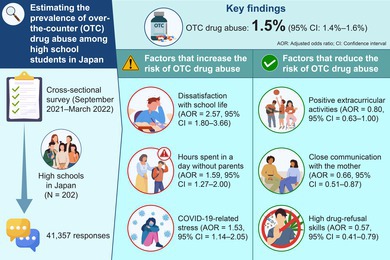

## 1 Introduction

Over‐the‐counter (OTC) drugs, purchased at pharmacies without a prescription, are familiar medicines in daily life. However, OTC drugs containing certain ingredients are abused, particularly by adolescents and young adults, and cause various health problems, such as acute intoxication and dependence [1, 2]. For example, cough and cold medicines containing dextromethorphan are typical OTC drugs that are abused [3, 4, 5]. The ingestion of high doses of dextromethorphan inhibits N‐methyl‐D‐aspartate (NMDA) receptors, causing hallucinogenic and dissociative effects [6, 7]. Cases of ingestion of high doses of dextromethorphan have reportedly resulted in assault, self‐harm, suicide, and murder [8]. This study focused on the abuse of OTC drugs, defined by the American College of Preventive Medicine as the self‐administration of medications to alter consciousness or “get high.” This definition guided our approach [9].

Studies on OTC drug abuse are limited compared to those on controlled substance abuse [10, 11, 12]. For example, studies in the UK [13] and Italy [14] reported OTC drug abuse prevalence, motivations, and risk factors among the general population. These reports provide useful information but have methodological shortcomings. In the study conducted in the UK, although random sampling was performed using the electoral register, the total sample size was 1000, and only about 400 participated in the survey. This small sample size affected representativeness. The Italian study had a larger sample size (approximately 700), but faced representativeness‐related issues due to recruiting participants through direct contact (e.g., acquaintances and students) and the Internet (e.g., emails, social media). Moreover, as these studies broadly targeted the general population, they were unable to obtain sufficient information on OTC drug abuse among adolescents and young adults.

In Japan, cases of OTC drug use disorders have risen significantly in recent years. According to a nationwide survey of psychiatric facilities, the proportion of such patients increased approximately six‐fold from 2012 to 2020 [15]. Secondary analysis of the survey data showed that these patients are predominantly young women with relatively high educational attainment and a low history of drug‐related crimes [16]. The most commonly abused OTC drugs include cough suppressants and cold medicines containing codeine or dihydrocodeine, consistent with trends in other countries. Additionally, painkillers containing bromovaleryl urea, which have been discontinued as medicines in many countries owing to intoxication and suicide risks, are still abused in Japan [17, 18].

Monitoring adolescent OTC drug abuse patterns through nationwide surveys is crucial for tackling adolescents' drug problems. Monitoring the Future (MTF), conducted by the University of Michigan in the United States, is the national dataset that has been reporting the prevalence of OTC drug abuse among school‐going adolescents since 2006 [19]. The MTF data show eighth graders' OTC drug abuse rose from 1.6% in 2015 to 4.6% in 2020, the highest since 2006, before declining to 3.2% in 2022; however, prevalence among tenth and twelfth graders remained stable.

To our knowledge, no nationwide epidemiological survey has been conducted on OTC drug abuse among adolescents in Asia, including Japan. Understanding adolescent OTC drug abuse requires assessing psychosocial factors, but such studies are less documented than those on illicit drug research. Therefore, this study aimed to examine the prevalence of OTC drug abuse among high school students in Japan and clarify the predictors of OTC drug abuse. This study is Asia's first large‐scale, random‐sample school survey on OTC drug abuse.

## 2 Methods

### 2.1 Study Design and Data Source

This study is a secondary analysis of the Nationwide High School Survey on Drug Use and Lifestyle 2021 [20]. This cross‐sectional survey examined Japanese high school students' current substance use and lifestyle statuses. This study was conducted at the National Center of Neurology and Psychiatry in Japan between September 2021 and March 2022 during the COVID‐19 pandemic.

The target population for the survey was 172 391 students enrolled in 202 high schools across the nation. The target schools were randomly selected through a stratified one‐stage community sampling method using six districts (Hokkaido‐Tohoku, Kanto, Hokuriku‐Tokai, Kinki, Chugoku‐Shikoku, and Kyushu‐Okinawa) as strata and each high school as a cluster. The inclusion criteria were regular high schools that held classes during the day, and the exclusion criteria were nighttime high schools that held classes at night and distance‐learning high schools that held classes online. Regular high school is a 3‐year educational program that mainly applies to students aged between 15 and 18. An anonymous self‐administered survey comprising 45 questions was conducted in high school classrooms. Questionnaires were distributed and collected by teachers at each school. Students completed the questionnaires in their classrooms, sealed them in individual envelopes, and submitted them to their teacher to minimize response bias.

The participants were informed in advance of the organization and purpose of the research, the voluntary nature of responses to the survey, and how the data would be used. The survey description was published on the Internet as a public notice and at the beginning of the questionnaire, which the teachers read before administering the survey. In this study, the informed consent procedure was omitted by guaranteeing participants the opportunity to refuse to participate. The study protocol was reviewed and approved by the Ethics Committee of the National Center of Neurology and Psychiatry (Approval no.: A2018‐055), and it conforms to the provisions of the Declaration of Helsinki.

### 2.2 Measures

#### 2.2.1 Abuse of OTC Drugs

Participants were asked about their history of OTC drug abuse over the past year, which was the primary outcome of this study. The types of OTC drugs used were cough medicines, cold medicines, and analgesics. OTC drug abuse was defined as using a drug in excess of the prescribed amount or frequency to get high or change one's mood. Internationally, the term “abuse” has become stigmatized and is avoided, particularly in the medical field. Japanese law (Pharmaceutical Affairs Law) [21] uses the Japanese word “Ranyo,” which means “abuse,” which is why the term “abuse” is used in this study; however, the term “abuse” was not used in expressions toward individuals.

#### 2.2.2 School Life‐Related Variables

In this study, variables related to school life, home life, and substance use were set as predictor variables based on previous studies on adolescent substance use. As a variable related to school life, this study asked participants about their level of satisfaction with school life, which was rated on a four‐point Likert scale ranging from “very satisfied” to “very dissatisfied.” This variable was based on reports that lower life satisfaction was associated with polysubstance use among high school students [22]. Additionally, this study examined the positive relationships between peers and extracurricular activities as protective factors against adolescent drug use [23]. The participants were asked to select one of four options based on their participation status: not participating, negatively participating, positively participating, or previously participated. Regarding positive relationships with peers [24], the following questions were asked: “Do you have close friends?” and “Do you have friends you can consult with?” with dichotomized options.

#### 2.2.3 Home Life‐Related Variables

As a variable related to home life, participants were asked about the average number of hours spent per day without their parents. This question was based on parental monitoring and support, which are protective factors against substance use among adolescents [25]. We instructed the participants to exclude time spent at school, cram school, extracurricular activities, and exercise when calculating the time. Participants were required to select one answer from five response options (none, less than 1 h, 1–2 h, 2–3 h, and more than 3 h). Furthermore, the participants were asked how often they consulted their mothers and fathers when they had problems, using a four‐point scale (never, rarely, sometimes, and often). This question aimed to evaluate adolescents' help‐seeking behavior from parents when they had problems. Although we did not provide specific examples of types of problems in the question, we assumed typical areas requiring help would include studying at school, friendships, and future plans. Given that this study was conducted during the pandemic, COVID‐19‐related stress was assessed as a variable related to home life [26]. The participants were asked the following question: “Looking back over the past year, how stressed do you feel about the schools being forced to close and having to stay at home due to the COVID‐19 pandemic?” Stress levels were evaluated using a four‐point scale (none, low, moderate, and high).

#### 2.2.4 Substance Use‐Related Variables

Participants were asked about their use of alcohol, tobacco, marijuana, organic solvents, methamphetamine, new psychoactive substances (NPS), cocaine, and MDMA within the past year. The substance types were based on survey items from a general population survey conducted in Japan [15]. Additionally, the participants were asked about their drug‐refusal skills, given that low refusal skills were a risk factor for substance use among adolescents [27, 28]. Drug refusal skills were assessed by examining self‐efficacy in refusing drugs. Participants responded to the question, “If a close friend or acquaintance encouraged you to use drugs, how confident are you in your ability to refuse?” using a four‐item Likert scale (none, low, moderate, and high).

### 2.3 Statistical Analysis

To examine the participants' characteristics, cross‐tabulations were performed with school location, area, grade, and age based on gender groups. The chi‐square test was used to verify significant differences between groups. In addition, to examine the bias among high school students across Japan, the national data were compared with the 2020 School Basic Survey [29], which was conducted following the Statistics Act of Japan, and significant differences were verified using a goodness‐of‐fit test.

The prevalence estimates of OTC drug abuse and other substance use were calculated by setting the probability of sampling students in each block as P1 and the probability of sampling survey respondents in each target school as P2. Weighting was calculated using the equation below; point estimates and standard errors were calculated.

$$\begin{array}{c} \text{P1}=\text{Number of students in target school}\\ /\text{number of high school students in each block} \end{array}$$

$$\begin{array}{c} \text{P2}=\text{Number of students}\;\mathrm{who}\;\text{responded to the survey}\\ /\text{number of students in target schools weight}\\ =\left(1/\text{p1}\right)\times \left(1/\text{p2}\right) \end{array}$$

Multivariable logistic regression models were used to examine the risk of OTC drug abuse in relation to the potential predictors. OTC drug abuse within the past year was set as the dependent variable and items related to students' school and home life as explanatory variables. Variables reported as risk or protective factors for adolescent drug use in previous studies [22, 23, 24, 25, 26, 27, 28] were included in the models. Demographic variables such as gender, age, and substance use other than OTC drugs were also included in the model as possible confounders. When entering explanatory variables into the simultaneous logistic regression model, we confirmed that the number of events per variable was 10 or more, following the criterion set in previous studies. Variance inflation factor (VIF) was used to check for multicollinearity, and the Hosmer–Lemeshow test was used to check the goodness of fit of the model. Adjusted odds ratios (AORs) and 95% confidence intervals were calculated using logistic regression analysis. Statistical analyses were conducted using IBM SPSS ver. 29.

## 3 Results

### 3.1 Sample Demographics

In total, 44 789 students from 80 high schools responded to the self‐administered questionnaire. The response rate was 39.6% for the target schools and 26.0% for the students. A total of 3432 students lacking data were excluded, and the remaining 41 357 valid responses were used in this study. The demographic characteristics of the participants are presented in Table 1. Regarding participants' gender, 52.3% were females, 46.7% were males, and 1.0% were others or unknown. The most common geographic location of the high schools where the survey was conducted was the Kanto region (26.6%), followed by the Hokkaido and Tohoku regions (11.0%). The most common age group was 16 years old (36.8%), followed by 17 (32.2%) and 18 years old (19.3%). Significant differences were observed between the participants' gender, location of high school (*p* < 0.001), and age (*p* < 0.001); however, no significant difference was observed between gender and grade (*p* = 0.436). When comparing this study's data with the national data, significant differences were observed in high school location (*p* < 0.001) and subject grade level (*p* < 0.001).

**TABLE 1:** Characteristics of survey responses by gender.

Characteristic	Total (*n* = 41 357)	Male (*n* = 19 321)	Female (*n* = 21 630)	Other or unknown (*n* = 406)	*p* ^a^	National data^b^	*p* ^c^
Location of the high schools					< 0.001		< 0.001
Hokkaido and Tohoku	11.0	11.8	10.3	12.1		10.9	
Kanto	26.6	24.1	29.0	24.9		34.7	
Hokuriku and Tokai	18.2	20.3	16.3	16.0		16.6	
Kinki	16.4	15.3	17.2	20.0		16.6	
Chugoku and Shikoku	12.7	12.8	12.7	9.4		9.0	
Kyushu and Okinawa	15.1	15.6	14.6	17.7		12.2	
Grade					0.436		< 0.001
First grade	39.3	39.4	39.1	41.1		33.4	
Second grade	32.2	32.4	32.1	33.5		33.3	
Third grade	28.5	28.2	28.8	24.5		33.3	
Age					< 0.001		
15	11.5	11.1	11.9	10.8			
16	36.8	37.0	36.6	37.7			
17	32.2	32.3	32.2	31.3			
18	19.3	19.4	19.3	17.2			
≥ 19	0.1	0.1	0.1	3.0			

^a^ *p*‐value for *χ* ^2^ test.

^b^ School Basic Survey 2020 based on the Statistics Act.

^c^ *p*‐value for *χ* ^2^‐test of goodness of fit.

### 3.2 Prevalence of OTC Drug Abuse and Substance Use

The estimated prevalence of OTC drug abuse, alcohol use, and cannabis use over the previous year is presented in Table 2. The prevalence of OTC drug use among the participants was 1.5%, that of alcohol was 12.5%, and that of marijuana was 0.1%. The prevalence of OTC drug abuse varied across subgroups. Regarding gender, the highest prevalences of OTC drug abuse were observed among “other” genders (8.3%), followed by females (1.7%) and males (1.2%). Regarding age, the highest prevalence was observed among ≥ 19 years (8.0%), followed by 15 years old (1.9%). No significant differences were observed regarding grades.

**TABLE 2:** Prevalence of over‐the‐counter drug abuse and substance use.

Characteristic	Past‐year, % (SE)		
	OTC drugs	Alcohol	Marijuana
Total	1.5 (0.1)	12.5 (0.7)	0.1 (0.0)
Gender			
Male	1.2 (0.1)	14.2 (0.8)	0.1 (0.0)
Female	1.7 (0.2)	10.8 (0.9)	0.1 (0.0)
Other or unknown	8.3 (1.7)	23.4 (2.9)	3.3 (1.4)
*p* ^a^	< 0.001	< 0.001	< 0.001
Grade			
First grade	1.5 (0.2)	9.5 (0.9)	0.1 (0.0)
Second grade	1.6 (0.2)	13.6 (0.8)	0.1 (0.0)
Third grade	1.7 (0.2)	16.8 (1.1)	0.3 (0.1)
*p* ^a^	0.242	< 0.001	< 0.001
Age			
15	1.9 (0.4)	9.2 (1.0)	0.1 (0.1)
16	1.3 (0.1)	10.2 (0.7)	0.1 (0.1)
17	1.6 (0.2)	14.5 (0.9)	0.2 (0.0)
18	1.6 (0.2)	17.1 (1.2)	0.2 (0.1)
≥ 19	8.0 (3.4)	45.6 (6.4)	1.0 (0.8)
*p* ^a^	< 0.001	< 0.001	< 0.001

*Note:* Estimate based on weighted data.

Abbreviations: OTC, over the counter; SE, standard error.

^a^ *p*‐value for Fisher's exact test.

The prevalence of alcohol use varied by gender; the prevalence was the highest among “other” genders (23.4%) and lowest among females (10.8%). Additionally, the prevalence of alcohol use increased with the age of the participants (first graders = 9.5%, second graders = 13.6%, and third graders = 16.8%); the prevalence was particularly high among participants aged ≥ 19 (45.6%).

Moreover, the prevalence of cannabis use also varied by gender; the highest prevalence was among “other” genders (3.3%) and equal among males (0.1%) and females (0.1%). Similar to the increase in the prevalence of alcohol use, an increase in the prevalence of cannabis use was observed with increased grades and age.

### 3.3 Predictors Associated With Self‐Reported Abuse of OTC Drugs

Table 3 presents the logistic regression analysis results, with OTC drug abuse over the past year as the dependent variable. VIF was employed to examine multicollinearity. NPS and cocaine use within the past year were excluded as variables from the logistic regression model because their VIF values exceeded 10. After excluding the two variables, the VIF was rechecked; the values of all remaining variables were below 10, indicating no multicollinearity. The *p*‐value of the Hosmer‐Lemeshow test was 0.463, confirming that the model was a good fit.

**TABLE 3:** Predictors of self‐reported abuse of over‐the‐counter drugs among high school students in Japan.

	Abused in the past year		Not abused in the past year		*p*	AOR	95% CI
	(*n* = 596)		(*n* = 40 761)				
	*n*	%	*n*	%			
Age							
15	76	1.6	4681	98.4		1.00	(reference)
16	206	1.4	15 014	98.6	0.128	0.81	0.62–1.06
17	189	1.4	13 140	98.6	0.052	0.76	0.57–1.00
18	118	1.5	7872	98.5	0.042	0.71	0.51–0.99
≥ 19	7	11.5	54	88.5	0.453	1.50	0.52–4.28
Gender							
Male	234	1.2	19 087	98.8		1.00	(reference)
Female	329	1.5	21 301	98.5	< 0.001	1.50	1.25–1.80
Others or unknown	33	8.1	373	91.9	< 0.001	3.17	2.01–5.00
Satisfaction with school life							
Very satisfied	184	1.1	16 612	98.9		1.00	(reference)
Satisfied	265	1.3	19 589	98.7	0.501	1.07	0.88–1.30
Dissatisfied	85	2.3	3630	97.7	0.009	1.45	1.10–1.92
Very dissatisfied	62	6.3	930	93.8	< 0.001	2.57	1.80–3.66
Close friends at school							
No	58	4.1	1340	95.9		1.00	(reference)
Yes	538	1.3	39 421	98.7	0.051	0.68	0.47–1.00
Consultation with a friend							
No	94	3.1	2958	96.9		1.00	(reference)
Yes	502	1.3	37 803	98.7	0.144	0.80	0.59–1.08
Extracurricular activities							
Not participating	143	1.8	7658	98.2		1.00	(reference)
Negative participating	81	2.2	3668	97.8	0.228	1.19	0.90–1.60
Positive participating	224	1.1	20 339	98.9	0.052	0.80	0.63–1.00
Previously participated	148	1.6	9096	98.4	0.683	1.05	0.82–1.36
Average hours per day without parents							
None	168	1.2	13 397	98.8		1.00	(reference)
Less than 1 h	91	1.3	6932	98.7	0.288	1.15	0.89–1.50
1–2 h	99	1.2	7998	98.8	0.870	1.02	0.79–1.32
2–3 h	66	1.2	5655	98.8	0.558	0.92	0.68–1.23
More than 3 h	172	2.5	6779	97.5	< 0.001	1.59	1.27–2.00
Consultation with father							
Never	278	1.6	17 657	98.4		1.00	(reference)
Seldom	92	1.2	7648	98.8	0.424	1.11	0.86–1.44
Sometimes	87	1.2	7032	98.8	0.355	1.14	0.87–1.48
Often	37	1.2	3104	98.8	0.459	1.15	0.79–1.69
No father	102	1.9	5320	98.1	0.164	1.19	0.93–1.51
Consultation with mother							
Never	191	2.1	9124	97.9		1.00	(reference)
Seldom	87	1.6	5415	98.4	0.409	0.89	0.67–1.18
Sometimes	168	1.2	14 101	98.8	0.005	0.71	0.56–0.90
Often	129	1.1	11 360	98.9	0.003	0.66	0.51–0.87
No mother	21	2.7	761	97.3	0.711	0.90	0.52–1.56
COVID‐19 related stress							
None	75	1.4	5472	98.6		1.00	(reference)
Low	112	1.2	9622	98.8	0.775	1.05	0.77–1.43
Moderate	231	1.3	17 490	98.7	0.174	1.22	0.92–1.61
High	178	2.1	8177	97.9	0.004	1.53	1.14–2.05
Drug‐refusal skills							
None	53	3.0	1716	97.0		1.00	(reference)
Low	44	5.6	735	94.4	0.007	1.88	1.19–2.97
Moderate	140	2.1	6435	97.9	0.972	0.99	0.69–1.42
High	359	1.1	31 875	98.9	< 0.001	0.57	0.41–0.79
Substance use within the past year							
Alcohol	186	3.6	4984	96.4	< 0.001	2.18	1.78–2.67
Tobacco	60	12.4	423	87.6	< 0.001	2.46	1.68–3.62
Cannabis	30	54.5	25	45.5	0.003	4.11	1.61–10.49
Organic solvents	22	56.4	17	43.6	0.057	6.03	0.95–38.19
Methamphetamine	19	67.9	9	32.1	0.779	0.67	0.04–11.44
MDMA	21	65.6	11	34.4	0.159	4.12	0.57–29.63

Abbreviations: AOR, adjusted odds ratio; CI, confidence interval.

Regarding demographic variables, the risk of OTC drug abuse was significantly higher among females (AOR = 1.50; 95% CI = 1.25–1.80) and “other” genders (AOR = 3.17; 95% CI = 2.01–5.00) than among males. Regarding age, the risk of OTC drug abuse was significantly lower among 18‐year‐olds (AOR = 0.71; 95% CI = 0.51–0.99) than among 15‐year‐olds.

Regarding school‐related outcomes, a significant association was observed between satisfaction with school life and the risk of OTC drug abuse. The students who responded “dissatisfied” (AOR = 1.45; 95% CI = 1.10–1.92) or “very dissatisfied” (AOR = 2.57; 95% CI = 1.80–3.66) were at significantly higher risk of abuse than those who responded “very satisfied.” In addition, a significant association was observed between extracurricular activities and OTC drug abuse; students who positively participated in extracurricular activities had a significantly lower risk of abusing OTC drugs (AOR = 0.80; 95% CI = 0.63–1.00) than those who did not participate. However, no significant association was observed between having close friends or friends to talk to and the risk of OTC drug abuse.

Regarding the home life‐related results, a significant association was observed between hours spent in a day without parents and the risk of OTC drug abuse. Compared with students who reported “never” spending time without their parents, students who reported being without their parents for “three or more hours per day” (AOR = 1.59; 95% CI = 1.27–2.00) had a significantly higher risk of OTC drug abuse. Additionally, the participants were asked how often they consulted their parents as an index of communication with their parents. Students who consulted their mothers more frequently were at lower risk of OTC drug abuse: “sometimes” (AOR = 0.71; 95% CI = 0.56–0.90) and “often” (AOR = 0.66; 95% CI = 0.51–0.87). However, no significant association was observed with the frequency of paternal consultations. Regarding COVID‐19‐related stress, students who reported “high” COVID‐19‐related stress (AOR = 1.53; 95% CI = 1.15–2.05) had a significantly higher risk of abusing OTC drugs than students who reported “no” stress.

Finally, regarding substance use‐related results, significant increases in OTC drug abuse risk were observed for alcohol (AOR = 2.18; 95% CI = 1.78–2.67), tobacco (AOR = 2.46; 95% CI = 1.68–3.62), and cannabis (AOR = 4.11; 95% CI = 1.61–10.49); however, no significant associations were observed for organic solvents, methamphetamine, and MDMA. Regarding drug‐refusal skills, students who responded “high” (AOR = 0.57; 95% CI = 0.41–0.79) were at significantly lower risk of OTC drug abuse than those who responded “none.”

## 4 Discussion

### 4.1 Prevalence of OTC Drug Abuse

Based on the rapid increase in patients with OTC drug use disorders, such as abusing cough and cold medicines, at psychiatric facilities in Japan, this study aimed to explore the prevalence of OTC drug abuse among high school students nationwide and examine the psychosocial characteristics of students with a history of OTC drug abuse. The prevalence of OTC drug abuse among Japanese high school students over the past year was estimated to be approximately 1.5%, that is, approximately one in 60 students, suggesting that the OTC drug abuse problem is widespread throughout Japan.

In this study, the prevalence of OTC drug abuse did not increase with age and was found to be highest at age 15. Contrarily, the prevalence of alcohol and marijuana use increased with age. One explanation for these contradictory results could be the different legal regulations for each substance. Young 15‐year‐old high school students may have fewer barriers to obtaining OTC drugs, which are not illegal, than alcohol and marijuana, which are illegal and prohibited by law in Japan.

### 4.2 Findings Related to Gender Difference

This study revealed several findings regarding the psychosocial characteristics of high school students who abused OTC drugs. First, a gender difference was observed in the prevalence of OTC drug abuse, with females having a significantly higher prevalence of OTC drug abuse than males. Previous studies on gender differences in substance use during adolescence found that females who experience disrupted parent–child relationships and chaotic home environments are more likely to use drugs than males in similar environments [30]. Additionally, studies on adolescents receiving substance abuse treatment suggest that females may need stronger and larger doses of drugs than males to cope with their traumatic symptoms [31]. These studies indicate that stress and trauma symptoms underlying OTC drug abuse are more prevalent in females, aiding interpretation of the current findings.

Additionally, regarding gender, this study found that students who identified as “unknown” or “other” had an even higher prevalence of OTC drug abuse than females. Students who responded to the gender question as “other” or “unknown” were more likely to be identified as sexual minorities. These results may suggest that students from sexual minority backgrounds experience more life stress and psychological trauma than heterosexual students. Previous studies examining the relationship between sexual orientation and adolescent substance use reported that LGBT youth have significantly higher rates of drug use than heterosexual youth [32], which assists in explaining this study's results.

### 4.3 Findings Related to Risk Factors

Second, regarding the risk factors for OTC drug abuse, the results indicated that high school students who were dissatisfied with their school life were at a higher risk of OTC drug abuse. Previous studies found that adolescents who withdrew from their school network and have older friends outside school are at a higher risk of drug use and that socially indifferent young individuals are more vulnerable to drinking and smoking than sociable young individuals [33, 34]. This study's results suggest that Japanese high school students who abuse OTC drugs are isolated in their school life and that withdrawal from their school network, including their friendships, may increase the risk of OTC drug abuse.

Moreover, this study found that high school students who spent more time without their parents at home were at a higher risk of OTC drug abuse. This may be related to the preventive effects of parental supervision. For example, a longitudinal study that followed a large group of adolescents aged 12–23 years reported that parental supervision was a predictor of drug use during early adolescence [25]. This study assessed parental supervision by asking the participants how much time they spent without a guardian. The relationship between parental monitoring and adolescent substance use risk is sometimes discordant between child and parental reports. However, positive family management predicts substance use only through child reports and not parental reports [35]. Given these previous findings, this study suggests that high school students' assessment of parental monitoring may predict the risk of OTC drug abuse.

Furthermore, the results indicated that high school students with high levels of COVID‐19‐related stress were at a significantly higher risk of OTC drug abuse. Previous studies found that drug use significantly increased during the pandemic and that drug use can be considered as a way to cope with pandemic‐related anxiety [26]. This study was conducted in 2021, when many young individuals, including high school students, were required to abstain from schools and stay at home as a preventive measure against COVID‐19. This study's findings suggest that stress and loneliness may have contributed to the increased risk of OTC drug abuse among high school students during the COVID‐19 pandemic.

### 4.4 Findings Related to Protective Factors

Third, regarding protective factors, this study found that the risk of abusing OTC drugs is significantly reduced when students participate in extracurricular activities positively. Previous studies found that school‐based extracurricular activities such as physical, cultural, and artistic activities provide opportunities for adolescents to enhance their strength and build skills with their peers [23, 36]. The results suggested that bonding with peers through extracurricular activities reduces feelings of isolation and acts as a protective factor against OTC drug abuse, which was consistent with previous studies [23].

Regarding the parent–child relationship, this study found that the more frequently children consulted their mothers about their worries, the lower the risk of OTC drug abuse. In contrast, no significant association was found between the frequency of consultations with fathers and OTC drug abuse. Research on adolescent substance use and parent–child relationships highlights gender‐specific variations in communication with fathers and mothers [37]. Moreover, a follow‐up study of junior high school students in Hong Kong reported that the mother–child relationship during adolescence was the most important and robust long‐term predictor of drug use [38]. The results indicate that the mother–child relationship during adolescence has a greater influence on drug use behavior than the father–child relationship, and these results suggest that a close relationship with one's mother may be a protective factor against OTC drug abuse among high school students.

Furthermore, this study assessed refusal self‐efficacy using a Likert scale, and the results indicated that students with high self‐efficacy had a significantly lower risk of OTC drug abuse than those with low self‐efficacy. Although it has been pointed out that research results regarding the relationship between adolescent drug use and low refusal skills are inconsistent [39], this study's results suggest that improving drug‐refusal skills may reduce the risk of substance use. Preventive education in schools is one of the most important measures against adolescent drug problems, and refusal skills in substance use are among the most important social skills during adolescence [40]. Systematic reviews on the effectiveness of school‐based prevention programs reported that programs focusing on social skills are more effective for prevention than regular curricula [41].

In Japan, drug abuse prevention education is part of the elementary to high school curriculum and is predominantly taught in health and physical education classes. However, the Japanese curriculum mainly focuses on illicit drugs, such as methamphetamine and marijuana, and does not include the prevention of OTC drug abuse. This study found that the prevalence of OTC drug abuse among high school students was significantly higher than that of marijuana, the most commonly abused controlled substance. Therefore, the educational curriculum does not match the actual situation of drug abuse among the youth. Consequently, the educational curriculum requires review, and education on use disorders and acute poisoning due to OTC drug abuse should be included.

### 4.5 Limitations of This Study

This study has some potential limitations that require caution when interpreting the results. The first is regarding the data's representativeness. This study randomly selected schools from all over Japan; however, only regular high schools with daytime classes were included. Therefore, students attending nighttime and online high schools were not included. Previous studies reported that the prevalence of alcohol, smoking, and marijuana use among nighttime school students was significantly higher than that among regular high school students [42]. In addition, nighttime school students were more likely to have experienced bullying or been absent from school than regular high school students [43]. Based on these previous studies, the actual prevalence of OTC drug abuse among high school students, including nighttime and online high schools, may be higher than the results of this study. In addition, the response rate in this study was low, and the data obtained may not represent all high school students nationwide. The low response rate is believed to be owing to the survey being conducted during the period when a state of emergency was declared due to the COVID‐19 pandemic, and schools did not have sufficient capacity to accept surveys from outside.

Second, this was a cross‐sectional study. Although this study examined the association between OTC drug abuse and various psychosocial factors among high school students, the findings did not necessarily prove causal relationships among the variables. Future follow‐up studies with a time course are required to prove causal relationships among the variables.

Third, the primary outcome of this study, the abuse of OTC drugs, was only assessed using self‐reporting. In addition, this study only collected information about drug abuse within the past year and did not provide detailed information such as the frequency of abuse, dosage per use, or specific brand names or ingredients. Self‐reported assessments of drug use are more prone to under‐reporting bias than assessments based on biological markers such as urine or hair tests [44, 45]. However, self‐reported underreporting is more likely to be influenced by recent experiences, such as drug use within 72 h, and by assessments of substances that are more stigmatized, such as drug offenses [46]. This study assessed self‐reported experiences of abuse within the past year, and there were no legal prohibitions against the abuse of OTC drugs. Therefore, an underreporting bias may have occurred in this study owing to self‐reports of OTC drug abuse; however, the degree of bias can be considered relatively small.

### 4.6 Clinical Implications

Despite these limitations, this study is the first in Asia to use nationwide data, based on random sampling, to estimate the prevalence of OTC drug abuse among high school students and to identify predictors related to their lifestyle. Therefore, this study has important public health policy implications. The findings may be useful in examining how OTC drugs are marketed to adolescents and in developing effective prevention education programs in schools. Furthermore, regarding the implications for psychiatric clinical practice, understanding the risk factors identified in this study may assist psychiatrists and pediatricians in recognizing early relapse risk in adolescents with OTC drug abuse problems. However, understanding the protective factors may help strengthen support for adolescents and their families connected to drug addiction treatment.

## 5 Conclusions

The prevalence of OTC drug abuse in the past year among Japanese high school students was estimated to be approximately 1.5%. This corresponds to approximately one in 60 high school students, suggesting that the OTC drug abuse problem is widespread throughout Japan. Isolation in both school and home may be a risk factor for OTC drug abuse among adolescents, whereas connections with parents and friends may have protective effects. Therefore, it is necessary to develop effective educational and treatment programs for OTC drug abuse among adolescents.

## Author Contributions

T.S. and S.I. designed the basic nationwide survey. T.M. obtained funding. T.S., S.I., K.K, and K.T. designed the questionnaire. T.S., S.I., and M.K. recruited participants, collected data, and created the database. T.S. conceived the study, performed the statistical analyses, interpreted the results, and wrote the manuscript. All authors revised and contributed to the final version of the manuscript. All authors have read and approved the final manuscript for publication.

## Ethics Statement

This study followed Japan's Ethical Guidelines for Medical and Biological Research Involving Human Subjects and conformed to the provisions of the Declaration of Helsinki. The study protocol was reviewed and approved by the Ethics Committee of the National Center of Neurology and Psychiatry (Approval no.: A2018‐055).

## Consent

The participants were informed in advance of the organization and purpose of the research, the voluntary nature of responses to the survey, and how the data would be used. The survey description was published on the Internet as a public notice and at the beginning of the questionnaire, which the teachers read before administering the survey. In this study, the informed consent procedure was omitted because the participants had the opportunity to refuse to participate.

## Conflicts of Interest

The authors declare no conflicts of interest.

## Data Availability

This study's dataset cannot be made publicly available, because data‐sharing approval has not been obtained from the Ethics Committee of the National Center of Neurology and Psychiatry. However, while raw data are not available, all the survey questionnaires, descriptive statistics for all the variables, cross‐tabulation results (by gender and grade level), and estimation‐related results are publicly accessible on the first author's institutional website (https://www.ncnp.go.jp/nimh/yakubutsu/report/pdf/highschool2021_ver2.pdf).
